# The reproductive season of *Acropora* in Socotra, Yemen

**DOI:** 10.12688/f1000research.3846.2

**Published:** 2014-04-09

**Authors:** Andrew H. Baird, David Abrego, Emily J. Howells, Vivian R. Cumbo

**Affiliations:** 1 ARC Centre of Excellence for Coral Reef Studies, James Cook University, Townsville, QLD 4811, Australia; 2 Department of Natural Science and Public Health, Zayed University, Abu Dhabi, United Arab Emirates; 3 New York University, Abu Dhabi, United Arab Emirates

## Abstract

Determining when corals reproduce has clear management and economic implications. Here we document the reproductive condition of corals in the genus *Acropora* on the island of Socotra in Yemen during February 2014. Twenty percent of colonies (n = 143) contained mature gametes and 28% had immature gametes indicating that spawning will occur in both February and March in 2014, confirming previous anecdotal reports of coral spawning at this time in Socotra. *Acropora* typically reproduce in synchrony with many other broadcast spawning scleractinian corals, and we therefore predict that many other species are reproductively active at this time of year.

## Observation

Most hermatypic scleractinian corals have an annual gametogenic cycle that culminates in the broadcast spawning of gametes once per year ^1^. In most reef regions, numerous species spawn in sychrony following full moons when sea surface temperature is either rising or falling ^2, 3^. Determining exactly when spawning takes place has important implications for reef management and clear economic benefits ^4^. For example, activities that are likely to limit fertilization success, such as dredging, can be prohibited during these often brief spawning periods ^5^. In addition, the diving industry can benefit from public interest in coral spawning.

**Figure 1.**
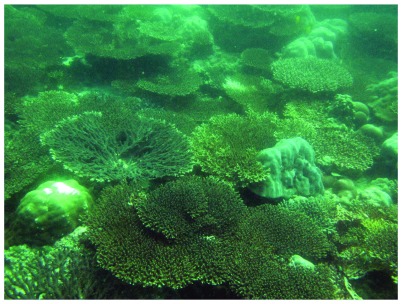
The island of Socotra supports diverse assemblages of *Acropora* species.

Here, we document the reproductive condition of *Acropora* corals on the island of Socotra, Republic of Yemen. The island, located 240 km east of the Horn of Africa and 380 km south of the Arabian Peninsula, supports a diverse scleractinian fauna of over 250 species (including 20 *Acropora* species) ^6^, and includes sites with a high cover of *Acropora* ( Figure 1). *Acropora* colonies were sampled before the full moon on 15 February 2014 to determine their reproductive condition. Three reproductive conditions were defined based on the appearance of the oocytes as observed with the naked eye in the field ^7^ (1) mature - oocytes pigmented and therefore likely to spawn within a month (2) immature - oocytes pale but visible indicating that they are close to maturity and likely to spawn within two to three months (3) empty - oocytes too small to see or absent indicating either that the colony has recently spawned, or is unlikely to do so for at least three months.

**Figure 2.**
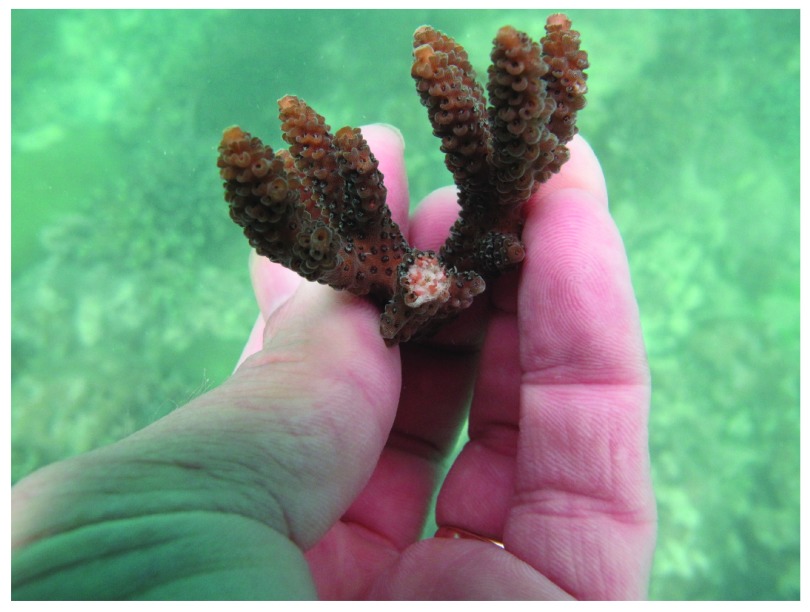
Mature (colored) oocytes are clearly visible in the branches of *Acropora* colonies.

A total of 143 *Acropora* colonies from approximately 14 species were sampled at four sites on the north of Socotra (Samerhur 12°41'40.96"N, 53°29'3.69"E; Qaiso 12°39'58.91"N, 53°24'33.86"E; Dihamri 12°40'20.35"N, 54°11'39.96"E; Hadibo 12°40'0.77"N, 54° 3'7.74"E) between the 31 January and 8 February 2014 ( Table 1). Twenty percent of colonies contained mature oocytes ( Figure 2), 28% contained immature oocytes and no oocytes were visible in the remaining 52% of colonies ( Table 1). Colonies with mature oocytes are highly likely to spawn at some time around the full moon in February 2014, whereas colonies with immature oocytes are likely to spawn in March 2014. The remaining colonies have either spawned recently, or alternatively, there could be a second reproductive season later in the year, similar to Western Australia ^8^, Singapore ^9^ and some locations in Indonesia ^1^. Ten species had at least one colony with mature gametes ( Table 1) suggesting a multi-species spawning event is likely in February 2014. The *Acropora* typically reproduce at much the same time as most other broadcast spawning scleractinian corals ^10, 11^ and therefore we predict that many other species will be spawning in February and March in Socotra. Our results confirm previous anecdotal reports of coral spawning on Socotra in February and March ^6^.

**Table 1.** The percentage of *Acropora* colonies with mature-, immature- or no oocytes, sampled between 31 January and 8 February 2014 on Socotra Yemen. n = number of sampled colonies.

Species	Percentage mature	Percentage immature	Percentage empty	n
*Acropora appressa*	100	0	0	1
*Acropora dendrum*	100	0	0	2
*Acropora downingi*	0	7	93	14
*Acropora cf humilis*	8	46	46	13
*Acropora lamarki*	33	33	33	3
*Acropora cf lutkeni*	33	33	33	12
*Acropora microphthalma*	0	0	100	2
*Acropora formosa*	0	11	89	18
*Acropora nasuta*	100	0	0	1
*Acropora roseni*	0	27	73	11
*Acropora cf solitaryensis*	67	33	0	3
*Acropora cf spicifera*	21	38	42	48
*Acropora valida*	44	33	22	9
*Acropora verweyi*	50	17	33	6
Total	20	28	52	143
