# Supplementary material for: The reproductive season of Acropora in Socotra, Yemen
Source: F1000Res. 2014 Apr 9;3:78. Originally published 2014 Mar 27. [Version 2] doi: 10.12688/f1000research.3846.2 (PMC4095572; doi:10.12688/f1000research.3846.2)
Supplement: Peer review history

# Peer review: Referee response for version 2

**DOI:** 10.5256/f1000research.4214.r4521

**Published:** 2014-04-17

**Authors:** Bert Hoeksema

**Affiliations:** 1 Department of Marine Zoology, Naturalis Biodiversity Center, Leiden, Netherlands

The authors followed up on my only recommendation. I have nothing more to add.

I have read this submission. I believe that I have an appropriate level of expertise to confirm that it is of an acceptable scientific standard.

# Peer review: Referee response for version 2

**DOI:** 10.5256/f1000research.4214.r4507

**Published:** 2014-04-17

**Authors:** Jean Kenyon

**Affiliations:** 1 Hawaiian and Pacific Islands National Wildlife Refuge Complex, US Fish and Wildlife Service, Honolulu, HI, USA

The authors have responded appropriately to my review comments, and to those of Bert Hoeksema. I am satisfied with this version of the article.

I have read this submission. I believe that I have an appropriate level of expertise to confirm that it is of an acceptable scientific standard.

# Peer review: Referee response for version 1

**DOI:** 10.5256/f1000research.4119.r4321

**Published:** 2014-04-01

**Authors:** Bert Hoeksema

**Affiliations:** 1 Department of Marine Zoology, Naturalis Biodiversity Center, Leiden, Netherlands

This short publication presents clear results and is well written. I have one comment. The conclusive sentence seems redundant, and may need rephrasing or some additional explanation if the authors want to maintain it. *“In addition, these data add to a growing body of evidence indicating that multi-specific spawning synchrony is a feature of all speciose coral assemblages.”*

The present results concern a selection of *Acropora* species. I wonder whether this would be sufficiently representative for a speciose coral assemblage. In theory any assemblage of two coral species can be considered multi-specific. With many *Acropora* species in a speciose coral assemblage, some degree of spawning synchrony is not surprising. So, in the present context I wonder if there is a minimum number of species for what counts as speciose and if the authors have a minimum number or percentage in mind for what exactly counts as multi-species spawning. If the authors cannot clarify their remark or cannot give more supportive information to this statement, I suggest that this sentence should be removed.

I have read this submission. I believe that I have an appropriate level of expertise to confirm that it is of an acceptable scientific standard.

# Response

**Published:** 2014-04-08

**Authors:** Andrew Baird

**Affiliations:** School of Marine Biology and Aquaculture, James Cook University, Australia

We have removed the sentence as suggested.

# Peer review: Referee response for version 1

**DOI:** 10.5256/f1000research.4119.r4277

**Published:** 2014-03-28

**Authors:** Jean Kenyon

**Affiliations:** 1 Hawaiian and Pacific Islands National Wildlife Refuge Complex, US Fish and Wildlife Service, Honolulu, HI, USA

Background information, purpose of study, data, and interpretation are all clearly and concisely presented. Documentation of coral sexual reproductive status at Socotra, Yemen in 14 species of *Acropora* provides a useful first contribution to improved understanding of global patterns of this reef maintenance and renewal process. Given that more than 250 species of scleractinian corals are supported at the island, it would be interesting and useful to extend these initial studies to additional species at other times of the year, and to confirm inferred spawning period and synchrony with field observations. 

That said, I would suggest that the article title, *“The reproductive season of scleractinian corals in Socotra, Yemen” *is too broad, given that only ~5% of the scleractinian fauna was sampled. A more focused title should indicate that the observations are confined to the genus *Acropora*. Within the article, it would also be informative to provide the number of species of *Acropora* reported among the scleractinian fauna, so as to give some perspective on the proportion of *Acropora *species sampled.

I have read this submission. I believe that I have an appropriate level of expertise to confirm that it is of an acceptable scientific standard.

# Response

**Published:** 2014-04-08

**Authors:** Andrew Baird

**Affiliations:** School of Marine Biology and Aquaculture, James Cook University, Australia

We have changed the title to *“The reproductive season of Acropora in Socotra, Yemen”.*

We have also provided the number of Acropora species reported to occur in Socotra as listed by Devantier *et al.,* 2004.
